# A Dual Swine Challenge With Porcine Circovirus Type 2 (PCV2) and *Mycoplasma hyopneumoniae* Used to Compare a Combination of Mixable Monovalent PCV2 and Monovalent *M. hyopneumoniae* Vaccines With a Ready-to Use PCV2 and *M. hyopneumoniae* Bivalent Vaccine

**DOI:** 10.3389/fvets.2020.00579

**Published:** 2020-09-02

**Authors:** Siyeon Yang, Taehwan Oh, Kee Hwan Park, Hyejean Cho, Chanhee Chae

**Affiliations:** Department of Veterinary Pathology, College of Veterinary Medicine, Gwanak-ro 1, Gwanak-gu, Seoul National University, Seoul, South Korea

**Keywords:** *Mycoplasma hyopneumoniae*, porcine circovirus type 2, porcine respiratory disease complex, vaccination, swine, bivlent vaccine, dual challenge

## Abstract

The present study evaluated the efficacy of swine vacciation using a combination of mixable monovalents for porcine circovirus type 2 (PCV2) and *Mycoplasma hyopneumoniae* against a ready-to-use bivalent vaccine under experimental conditions. Pigs at 21 days of age were administered either a combination of two mixable monovalent vaccines or a bivalent vaccine containing PCV2 and *M. hyopneumoniae*. Vaccination was followed with an *M. hyopneumoniae* challenge at 42 days of age (−14 days post challenge, dpc) and a PCV2d challenge at 56 days of age (0 dpc). Each vaccinated and challenged group was compared with the unvaccinated and challenged group for clinical, microbiological, immunologic, and pathologic differences. Clinically, two vaccinated and challenged groups showed minimal respiratory diseases that was characterized by occasionally coughing and sneezing. A significant difference was not calculated in the average daily weight gain, nasal shedding of *M. hyopneumoniae*, and pathological lesions between two vaccinated and challenged groups. A combination of two monovalent vaccines mixed into a combo prior to vaccination followed by challenge resulted in increased numbers of PCV2d-specific interferon-γ secreting cells at 21 dpc and a significant reduction in PCV2d viremia at 14 dpc when compared with the ready-to-use bivalent-vaccinated and challenged groups. These results offer supporting evidence that vaccination during the weaning to finishing period against *M. hyopneumoniae* and PCV2 is efficacious for controlling diseases caused by these two pathogens.

## Introduction

Porcine respiratory disease complex (PRDC) is one of the most important swine health concerns to producers today. PRDC is most prevalent in large swine farms that implement a continuous production system, often containing around 6–20 weeks-old pigs (1, 2). Common clinical symptoms of PRDC include growth retardation, decreased feed efficiency, anorexia, lethargy, fever, cough, and dyspnea.

Porcine circovirus type 2 (PCV2) is the smallest non-enveloped, single-stranded, circular DNA virus within the genus *Circovirus* and the family *Circoviridae* (3). Currently, PCV2 is classified with six different genotypes, a-f (4–7) with PCV2d prevailing as the most frequent genotype currently circulating within the US and Korea (8, 9). PCV2 is the causative agent of PCV2-systemic disease as well as various other circovirus-related syndromes (10–12).

*Mycoplasma hyopneumoniae* bacterium is one of the most important pathogens within the PRDC, and causes chronic respiratory conditions in pigs as the etiologic agent of enzootic pneumonia. Dry, non-productive coughing, and growth retardation are the most obvious clinical signs, although the majority of *M. hyopneumoniae* infections are subclinical (13).

PCV2 and *M. hyopneumoniae* are both known to affect swine herds worldwide. PRDC development is often caused by a co-infection of these two pathogens (14). This in turn has resulted in an increase in animal treatment costs as well as in economic losses. Vaccination within the Asian pork industry remains the most common and effective tool in controlling PCV2 and *M. hyopneumoniae* infection. The recommended times of vaccination against PCV2 and *M. hyopneumoniae* are similar so the use of either a combination of two monovalent vaccines mixed into a combo prior to vaccination, or a ready-to-use combination vaccine containing PCV2 and *M. hyopneumoniae* are growing in popularity. The objective of this study was to compare the efficacy of a combination of two monovalent vaccines mixed into a combo prior to vaccination with a ready-to-use bivalent vaccine containing PCV2 and *M. hyopneumoniae*. The vaccines were evaluated with a dual *M. hyopneumoniae* and PCV2d challenge in pigs and conclusions were based on clinical, microbiological, immunological, and pathological outcomes.

## Materials and Methods

### Ethical Statement

This study was approved by the Seoul National University Institutional Animal Care and Use Committee (SNU-190712-5).

### Animals

Piglets were selected from commercial farms which tested free of porcine reproductive and respiratory syndrome virus (PRRSV) and *M. hyopneumoniae* based on breeding herd serology, and long term clinical and slaughter history. A total of 60 colostrum-fed, cross-bred, conventional piglets were purchased at 7 days old from the selected facilities. Upon arrival, piglets were tested for PCV2 and PRRSV viremia as well as for nasal shedding of *M. hyopneumoniae* by real-time polymerase chain reaction (PCR). Piglets were also tested and confirmed seronegative for PCV2 (SERELISA PCV2 Ab Mono Blocking, Synbiotics, Lyon, France), PRRSV (HerdCheck PRRS X3 Ab test, IDEXX Laboratories Inc., Westbrook, ME, USA), and *M. hyopneumoniae* (*M*. *hyo*. Ab test, IDEXX Laboratories Inc.), according to routine serological testing.

### Experimental Design

The random number generator function (Excel, Microsoft Corporation, Redmond, Washington, USA) was used to randomly divide 60 pigs into 4 groups, each containing 15 pigs (Table 1). The minimum sample size per each group was calculated as suggested by Cohen (15) using pwr package in R v.3.5.1 (R Core Team: a language and environment for statistical computing. R Foundation for Statistical Computing, Vienna, Austria, http://www.r-project.org). The minimum number of piglets needed per group was calculated using a 0.05 significance level, 0.4 effect size, and 70% power. Under these parameters, the minimum number of pigs per group equated to 14.69, therefore, at least 15 piglets were designated per group. Three groups of pigs, identified as VacA/Ch, VacB/Ch, and UnVac/Ch, were randomly assigned into 3 rooms. Pigs in an additional UnVac/UnCh group were randomly assigned into 1 room. Each room uniformly constained 2 pens (7 or 8 pigs per pen) and each pig was assigned a pen randomly using the random number generator function (Excel, Microsoft Corporation). Rooms and pens were consistent in design and were equipped with equipped with free-access to water and feed throughout the duration of the study.

**Table 1:** Experimental design with vaccination and challenge strategies for *Mycoplasma hyopneumoniae* (Mhyo) and porcine circovirus type 2d (PCV2d) at different days post-challenge (dpc).

	**Vaccination (dpc)**	**Challenge (dpc)**		**Necropsy (dpc)**
**Groups**	**−35**	**−14**	**0**	**21**
Age (days)	21	42	56	77
VacA/Ch	FLEXcombo (CircoFLEX-MycoFLEX)	Mhyo	PCV2d	15 pigs
VacB/Ch	Porcilis PCV M Hyo	Mhyo	PCV2d	15 pigs
UnVac/Ch	None	Mhyo	PCV2d	15 pigs
UnVac/UnCh	None	None	None	15 pigs

*Different letters mean statistically significant differences within 5 groups (P < 0.05)*.

Pigs in the VacA/Ch group were vaccinated intramuscularly with FLEXcombo (Boehringer Ingelheim Vetmedica Inc., St. Joseph, Missouri, USA) at 21 days of age in accordance with the manufacturer's directions. FLEXcombo vaccine was prepared by mixing equal volumes of Ingelvac CiroFLEX (Serial No. 3091282A) and Ingelvac MycoFLEX (Serial No. 273052A). Each pigs in VacA/Ch group received a 2 mL/dose of freshly mixed FLEXcombo vaccine. Pigs in VacB/Ch group were intramuscularly vaccinated with a 2.0 mL dose of the ready-to-use combination vaccine Porcilis PCV M Hyo (Lot No. C530B01, MSD Animal Health, Boxmeer, Netherlands), also at 21 days of age. UnVac/Ch and UnVac/UnCh pig groups were administered a 2.0 mL dose of phosphate buffered saline (PBS, 0.01M, pH 7.4) at 21 days old.

Pigs in the VacA/Ch, VacB/Ch, and UnVac/Ch groups were anesthetized at −14 days post challenge (dpc, 42 days old), with a mixture of 2.2 mg/kg xylazine hydrochloride (Rumpon, Bayer Korea, Seoul, Korea), 2.2 mg/kg tiletamine hydrochloride, and 2.2 mg/kg zolazepam hydrochloride (Zoletil 50, Virbac Korea, Seoul, Korea) by intramuscular injection. Post-anesthetization, they were immediately inoculated with an intratracheal challenge containing 7 mL of *M. hyopneumoniae* culture medium with 10^7^ color changing units (CCU)/mL as described previously (16, 17). VacA/Ch, VacB/Ch, and UnVac/Ch pig groups were inoculated intranasally with 3 mL of tissue culture supernatant containing 1.2 × 10^5^ TCID_50_/mL of PCV2d (SNUVR140004, GenBank no. KJ437506) at 0 dpc (56 days old) (12).

Blood and nasal swabs were collected at the following time points: −49 (7 days old), −35 (21 days old), −7 (42 days old), 0 (56 days old), 7 (63 days old), 14 (70 days old), and 21 (77 days old) dpc. An intravenous injection of sodium pentobarbital was used to sedate all pigs prior to euthanization by electrocution at 21 dpc as previously described (18).

### Clinical Observations

Pigs were monitored daily for clinical signs and scored weekly using a score ranking system which ranged from 0 (normal) to 6 (severe dyspnea and abdominal breathing) (19). All observers involved in these processes were blinded to vaccination and type of vaccine status. At the end of the study, mortality rate was calculated for each group. Mortality rate was defined as the number of pigs that died divided by the number of pigs initially assigned to that group within the batch. All pigs that died or were culled throughout the study were necropsied.

### Average Daily Weight Gain

The live weight of each pig was measured at −35 dpc (21 days old), 0 dpc (56 days old), and 21 dpc (77 days old). Average daily weight gain (ADWG; grams/pig/day) was analyzed from the collected data over two time periods: (i) between 21 and 56 days of age and (ii) between 56 and 77 days of age. ADWG during these two production stages was then calculated as the difference between the starting and final weight divided by the duration of the stage. Data for dead or removed pigs were included in the calculation.

### Quantification of *M. hyopneumoniae* in Nasal Swabs

A commercial kit (QIAamp DNA Mini Kit, QIAGEN, Valencia, California, USA) was used to extract DNA from nasal swabs prior to quantification of *M. hyopneumoniae* genomic DNA copy numbers by real-time PCR (20).

### Quantification of PCV2d DNA in Blood

A commercial kit (QIAamp DNA Mini Kit, QIAGEN) was used to extract DNA from serum samples prior to quantification of PCV2 genomic DNA copy numbers by use of real-time PCR (21).

### Serology

All collected serum samples were tested using the commercially available *M. hyopneumoniae* (*M*. *hyo*. Ab test, IDEXX Laboratories Inc.) and PCV2 (SERELISA PCV2 Ab Mono Blocking, Synbiotics) ELISA kits in accordance with the manufacturer's instructions. Serum samples were considered positive for *M. hyopneumoniae* antibody if the sample-to-positive (S/P) ratio was ≥0.4, while serum samples were considered to be positive for anti-PCV2 antibody if the reciprocal ELISA titer was >350.

### Enzyme-Linked Immunospot (ELISPOT) Assay

The *M. hyopneumoniae* and PCV2d challenge strains were used in the *in vitro* stimulation of peripheral blood mononuclear cells (PBMC) so that the numbers of *M. hyopneumoniae*- and PCV2d-specific interferon-γ secreting cells (IFN-γ-SC) could be quantified (21, 22). All results were expressed as the numbers of IFN-γ-SC per million PBMC. The frequency of *M. hyopneumoniae*- and PCV2d-specific IFN-γ-SC was considered to be positive if the number of *M. hyopneumoniae*- and PCV2d-specific IFN-γ-SC was >20 cells/10^6^ PBMC.

### Pathology

Macroscopic lung lesion severity was scored to estimate the percentage of the lung affected by pneumonia (23).

Two veterinary pathologists (Chae and one graduate student) were blinded while examing lung and lymphoid tissue sections. Mycoplasmal pneumonia lesions were scored (0–6) based on the severity of peribronchiolar and perivascular lymphoid tissue hyperplasia (24). Interstitial pneumonia lesions were scored (0–6) based on the severity of interstitial pneumonia (19). PCV2-associated lymphoid lesions were scored (0–5) based on the severity of lymphoid depletion and granulomatous inflammation (10, 25).

### Statistical Analysis

Real-time PCR data were transformed to log_10_ values prior to statistical analysis. Data were tested for normal distribution using the Shapiro-Wilk test. One-way analysis of variance (ANOVA) was used to examine whether there were statistically significant differences among the four groups at each defined time point. Test results showing a statistical significance from one-way ANOVA were then subjected to a *post-hoc* test for a pairwise comparison with Tukey's adjustment. If the normality assumption was not met, the Kruskal-Wallis test was additionally performed. Any results showing statistical significance from the Kruskal Wallis test were then subjected to the Mann-Whitney test with Tukey's adjustment to compare the differences among the groups. A value of *P* < 0.05 was considered to be significant.

## Results

### Clinical Observation

**Figure 1:**
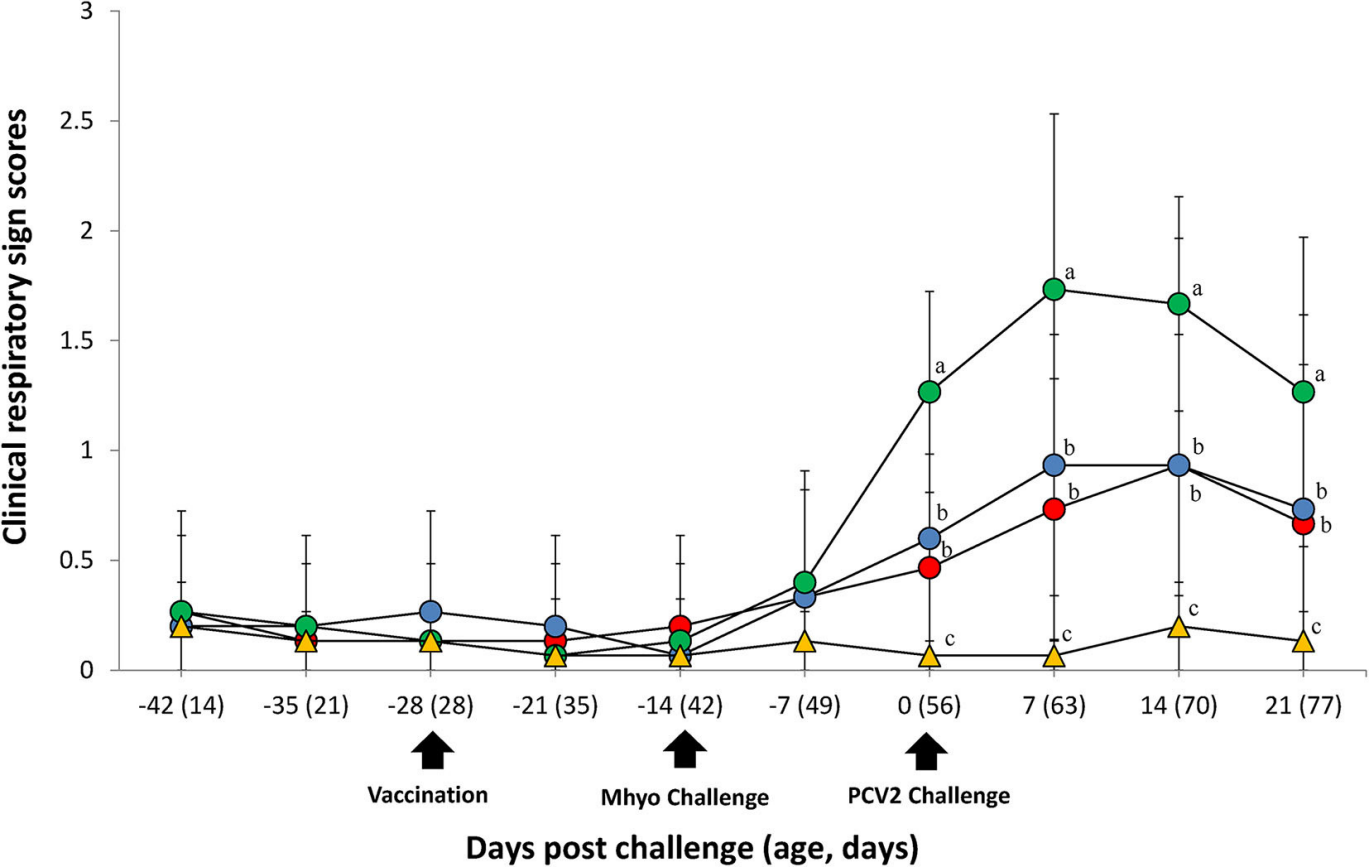
Clinical resipiratory sign. Clinical respiratory sign scores in the different groups: VacA/Ch (•), VacB/Ch (•), UnVac/Ch (•), and UnVac/UnCh (▴). Different letters within a sampling point mean statistically significant differences (*P* < 0.05). Variation is expressed as the standard deviation. Different letters within a sampling point mean statistically significant differences (*P* < 0.05).

In UnVac/Ch group pigs exhibited mild-to-moderate respiratory disease that was characterized by lethargy, coughing, and occasionally sneezing between 7 and 14 dpc. Moderate-to-severe respiratory disease that was characterized by coughing, depression, and pronounced abdominal breathing was also observed the same timeperiod. At 21 dpc, symptoms in the UnVaqc/Ch group pigs regressed to mild-to-moderate respiratory disease. Pigs in VacA/Ch and VacB/Ch groups showed minimal respiratory diseases that was characterized by occisionally coughing and sneezing. No respiratory diseases were observed in pigs from UnVac/UnCh group. The mean scores for respiratory disease from 0 to 21 dpc were signficantly higher (*P* < 0.05) in pigs from the UnVac/Ch group when compared with the VacA/Ch, VacB/Ch, and UnVac/UnCh groups (Figure 1).

### Average Daily Weight Gain

All 4 groups were similar in average body weights at the start of the study (21 days of age). Pigs from the VacA/Ch, VacB/Ch, and UnVac/UnCh groups had significantly higher (*P* < 0.05) ADWG between 56 and 77 days of age when compared with pigs in the UnVac/Ch group. The overall ADWG results (from 21 to 77 days old) of pigs from the VacA/Ch, VacB/Ch, and UnVac/UnCh groups was significantly higher (*P* < 0.05) when compared with in the UnVac/Ch group. A significant difference was not calculated in the ADWG among the VacA/Ch, VacB/Ch, and UnVac/UnCh groups (Table 2).

**Table 2:** Average daily weight gain (ADWG) in pigs from different group.

**Groups**	**Average Daily Weight Gain (grams/day/pig)**		
	**21–56**	**56–77**	**21–77**
VacA/Ch	321.90 ± 6.44	546.98 ±57.87^a^	406.30 ± 20.56^a^
VacB/Ch	319.80 ± 36.85	512.38 ± 75.65^a^	392.02 ± 48.16^a^
UnVac/Ch	315.61 ± 22.33	408.25 ± 22.44^b^	350.35 ± 17.48^b^
UnVac/UnCh	327.80 ± 16.74	581.90 ± 61.43^a^	423.09 ± 19.14^a^

*Different letters mean statistically significant differences within 5 groups (P < 0.05)*.

### Genomic Quantification of *M. hyopneumoniae* and PCV2

**Figure 2:**
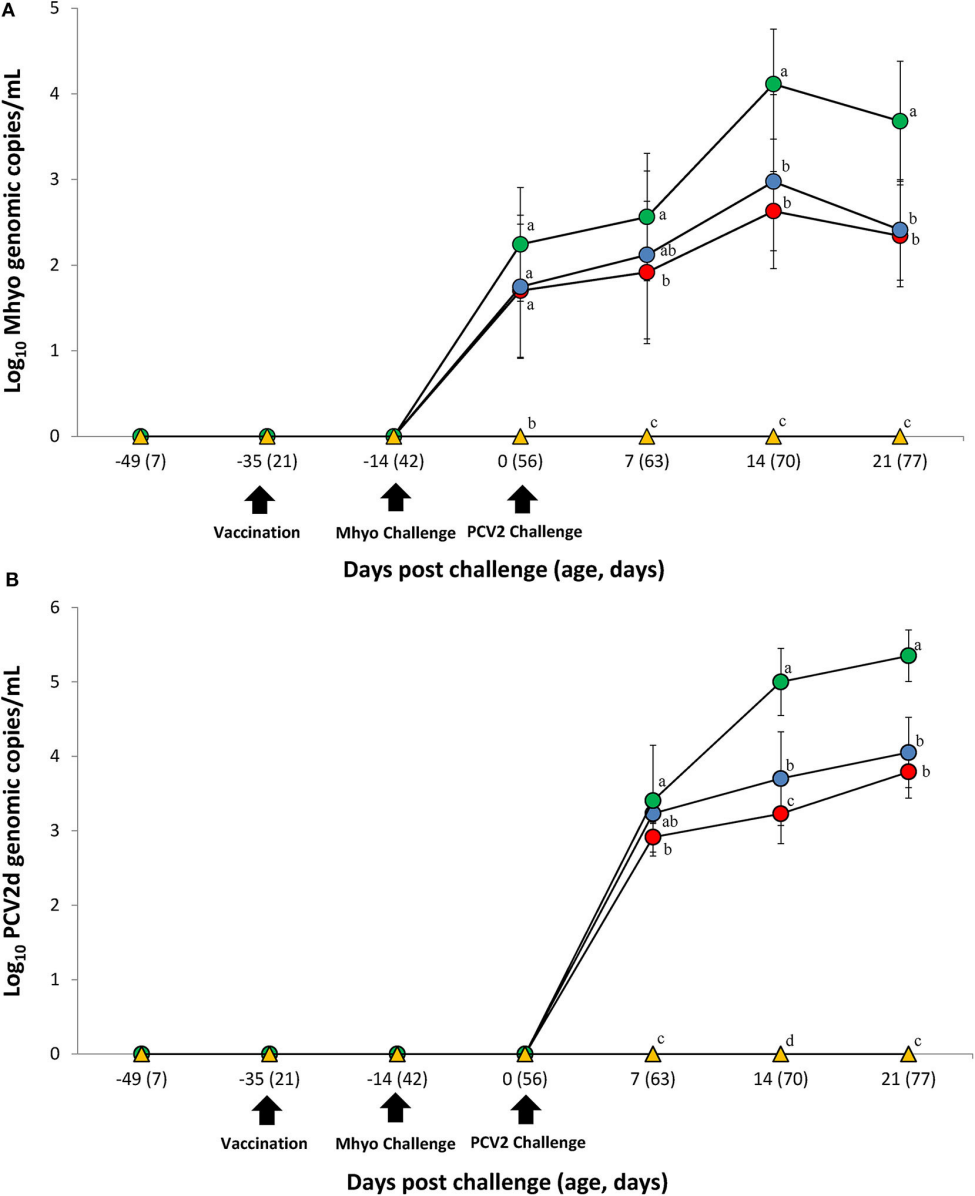
Genomic quantification. **(A)** Mean values of the genomic copy number of *Mycoplasma hyopneumoniae* (Mhyo) DNA in nasal swabs. **(B)** Mean values of the genomic copy number of porcine circoviorus type 2d (PCV2d) DNA in serum in the different groups: VacA/Ch (•), VacB/Ch (•), UnVac/Ch (•), and UnVac/UnCh (▴). Different letters within a sampling point mean statistically significant differences (*P* < 0.05). Variation is expressed as the standard deviation. Different letters within a sampling point mean statistically significant differences (*P* < 0.05).

Prior to challenge, zero genomic copies of *M. hyopneumoniae* and PCV2 were detected in any of the pigs within the study. For genomic quantification of *M. hyopneumoniae*, pigs in the VacA/Ch group had significantly less (*P* < 0.05) *M. hyopneumoniae* genomic copies in their nasal swabs at 7 dpc when compared with the UnVac/Ch group. Pigs in the VacA/Ch and VacB/Ch groups had significantly less (*P* < 0.05) *M. hyopneumoniae* genomic copies in their nasal swabs at 14 and 21 dpc when compared with the UnVac/Ch group (Figure 2A).

For genomic quantification of PCV2, pigs in the VacA/Ch group had a significantly lower (*P* < 0.05) number of genomic copies of PCV2 in their blood at 7 dpc when compared with the UnVac/Ch group. Pigs in the VacA/Ch and VacB/Ch groups had a significantly lower (*P* < 0.05) number of genomic copies of PCV2 in their blood at 14 and 21 dpc when compared with the UnVac/Ch group. Pigs in the VacA/Ch group had a significantly lower (*P* < 0.05) number of genomic copies of PCV2 in their blood at 14 dpc when compared with the VacB/Ch group. No genomic copies of *M. hyopneumoniae* or PCV2 were detected in any of the pigs from the UnVac/UnCh group (Figure 2B).

### Immune Responses Against *M. hyopneumoniae*

**Figure 3:**
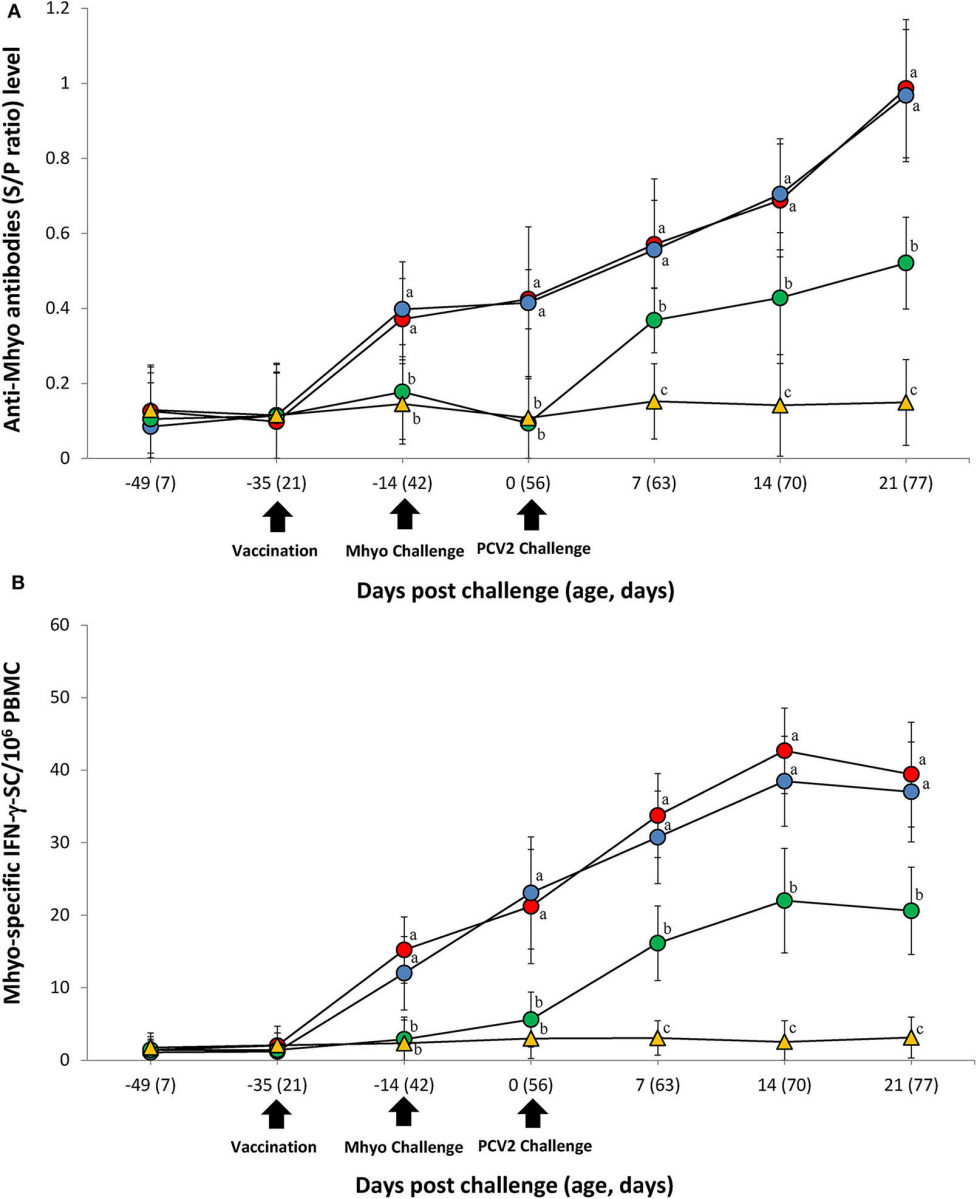
Immune responses against *Mycoplasma hyopneumoniae* (Mhyo). **(A)** Mean values of the Mhyo ELISA antibodies. **(B)** Frequency of Mhyo-specific interferon-γ secreting cells (IFN-γ-SC)/10^6^ peripheral blood mononuclear cells (PBMC) in the different groups: VacA/Ch (•), VacB/Ch (•), UnVac/Ch (•), and UnVac/UnCh (▴). Variation is expressed as the standard deviation. Different letters within a sampling point mean statistically significant differences (*P* < 0.05).

ELISA was used to assess pig antibody responses against *M. hyopneumoniae*. Pigs in the VacA/Ch and VacB/Ch groups had significantly higher (*P* < 0.05) *M. hyopneumoniae* ELISA S/P ratios between −14 and 21 dpc when compared with the UnVac/Ch groups. Antibodies against *M. hyopneumoniae* were not detected in any of the pigs from the UnVac/UnCh group (Figure 3A).

The number of *M. hyopneumoniae*-specific IFN-γ-SC quantified in the PBMC of individual pigs was used to categorize T cell response. Pigs from the VacA/Ch and VacB/Ch groups had a significantly higher (*P* < 0.05) number of *M. hyopneumoniae*-specific IFN-γ-SC in their PBMC between −14 and 21 dpc when compared with pigs from the UnVac/Ch group. The mean numbers of *M. hyopneumoniae*-specific IFN-γ-SC in the UnVac/UnCh group remained at basal levels (<20 cells/10^6^ PBMC) throughout the study (Figure 3B).

### Immune Responses Against Porcine Circovirus Type 2

**Figure 4:**
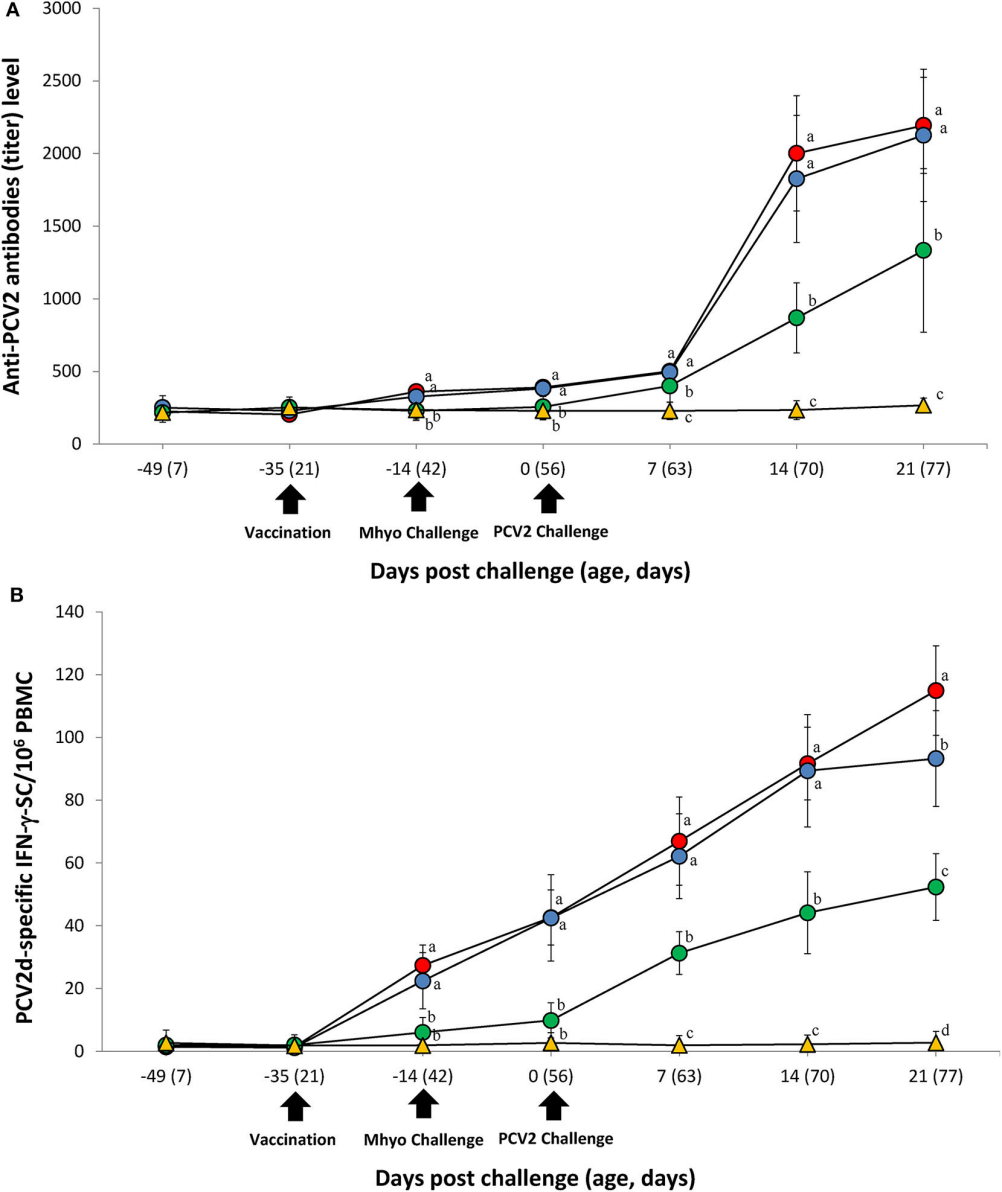
Immune responses against porcine circovirus type 2 (PCV2). **(A)** Mean values of the PCV2 ELISA antibodies. **(B)** Frequency of PCV2d-specific interferon-γ secreting cells (IFN-γ-SC)/10^6^ peripheral blood mononuclear cells (PBMC) in the different groups: VacA/Ch (•), VacB/Ch (•), UnVac/Ch (•), and UnVac/UnCh (▴). Variation is expressed as the standard deviation. Different letters within a sampling point mean statistically significant differences (*P* < 0.05).

ELISA was used to assess pig antibody responses PCV2. Pigs in the VacA/Ch and VacB/Ch groups had significantly higher (*P* < 0.05) PCV2 ELISA titers between −14 and 21 dpc when compared with those in the UnVac/Ch groups. Antibodies against PCV2 were not detected in any of the pigs from the UnVac/UnCh group (Figure 4A).

Pigs from the VacA/Ch and VacB/Ch groups had a significantly higher (*P* < 0.05) number of PCV2d-specific IFN-γ-SC in their PBMC between −14 and 21 dpc when compared with pigs in the UnVac/Ch group. Pigs from the VacA/Ch group had significantly higher (*P* < 0.05) numbers of PCV2d-specific IFN-γ-SC in their PBMC at 21 dpc when compared with pigs in the VacB/Ch group. The mean numbers of PCV2d-specific IFN-γ-SC in the UnVac/UnCh group remained at basal levels (<20 cells/10^6^ PBMC) throughout the study (Figure 4B).

### Pathology

Pigs in the VacA/Ch and VacB/Ch groups had significantly lower (*P* < 0.05) macroscopic lung lesion scores, microscopic mycoplasmal and interstitial lung lesion scores, and microscopic lymphoid lesions at 21 dpc when compared with pigs from the UnVac/Ch group. Macroscopic and microscopic lung lesions, and micriscopic lymphoid lesions were not observed in pigs from the UnVac/UnCh group (Table 3).

**Table 3:** Lung and lymphoid lesion scores in pigs from different groups.

	**Macroscopic**	**Microscopic**		
**Groups**	**Lung lesion score**	**Mycoplasmal lung lesion score**	**Interstitial lung lesion score**	**Lymphoid lesion score**
VacA/Ch	21.73 ± 6.84^b^	1.26 ± 0.39^b^	1.34 ± 0.32^b^	1.02 ± 0.24^b^
VacB/Ch	25.27 ± 6.34^b^	1.28 ± 0.48^b^	1.56 ± 0.71^b^	1.17 ± 0.19^b^
UnVac/Ch	53.20 ± 7.93^a^	3.06 ± 0.41^a^	2.84 ± 0.56^a^	2.62 ± 0.35^a^
UnVac/UnCh	5.8 ± 4.87^c^	0.09 ± 0.10^c^	0.19 ± 0.21^c^	0.2 ± 0.13^c^

*Different letters mean statistically significant differences within 5 groups (P < 0.05)*.

## Discussion

Both presentations of the evaluated vaccines (a combination of two monovalent vaccines mixed into a combo prior to vaccination, or a ready-to-use bivalent vaccine) proved efficacious in the protection of pigs against *M. hyopneumoniae* and PCV2d challenge. The study included a successful challenge infection as all infected pigs tested positive for *M. hyopneumoniae* and PCV2d by PCR testing of nasal swabs and blood, respectively. The present data demonstrated that the two most prominent and costly diseases, PCVAD and enzootic pneumonia, can be prevented with either form of a combination vaccine, two monovalents which are mixed onsite prior to vaccination (a combination of two monovalent vaccines mixed into a combo prior to vaccination) or a ready-to-use vaccine. Growth performance was the most critical parameter evaluated throughout the study and should be given preccedece when evaluating the efficacy of a vaccine (26). Vaccination and challenge by either method resulted in significant growth performance improvement when compared with the unvaccinated-challenged group. Although ADWG was numerically higher in the mixable combo-vaccinated group than in the ready-to-use bivalent-vaccinated group, it was not determined as statistically significant.

Field conditions were mimicked through the vaccination-infection model design of this study. Analysis of diagnostic cases and serological surveyance under Korean field conditions (C. Chae, personal observation) has concluded that pigs are generally prone to *M. hyopneumoniae* infection at 5–7 weeks of age and to PCV2 infection at 7–9 weeks of age, which eventually leads to a PRDC outbreak at 11–16 weeks of age. These natural-occurring infection timeframes were kept in mind when designing this study, which is why pigs were inoculated with *M. hyopneumoniae* at 6 weeks of age followed by PCV2 inoculation at 8 weeks of age. In this sequential infection model, *M. hyopneumoniae* infection followed by PCV2 reproduced PCVAD experimentally, mimicking natural cases in the field as described in the previous study (27). Vaccination of pigs with PCV2 and *M. hyopneumoniae* at 3 weeks of age provided statistically significant protection against *M. hyopneumoniae* at 6 weeks of age and against PCV2 at 8 weeks of age.

Large opportunities still exist to develop a better understooding of protective immunity against *M. hyopneumoniae*. As a non-invasive pathogen that can induce pneumonia, cell-mediated immune response must play a significant immulogical role. A consistent correlation between the induction of cell-mediated immunity and the severity of pneumonic lesions has been demonstrated (28, 29). A significant difference was not found between the two sets of vaccines used in this study, in terms of elicited cell-mediated immune response and protective effect against *M. hyopneumoniae* challenge. The strain of *M. hyopneumponiae* was identical in both sets of vaccines (VacA/Ch and VacB/Ch groups) but the vaccine adjuvants varied (30). Although adjuvant formulation in particular is known to affect the immunogenicity and protective effect of inactivated whole-cell *M. hyopneumoniae* bacterins (31), an effect on efficacy against *M. hyopneumoniae* was not observed in the present study.

Both vaccines evaluated in this study were based on the PCV2a genotype. PCV2d viremia was still reduced in pigs from PCV2a-based vaccination and PCV2d-challenge when compared with unvaccinated and challenged pigs. Reduction of PCV2d Viremia is clinically meaningful as PCV2d is currently the predominant PCV2 genotype circulating within Asian pig herds (8). The reduction of PCV2 viremia is well-correlated with protection against PCV2 infection (32–34). Both types of vaccines elicited the PCV2d-specific IFN-γ-SC which is critical in reducing the amount of PCV2 viremia within pigs (35–37). PCV2d viremia and lymphoid lesion reduction is both attributed to the induction of protective cell-mediated imminuty. The two vaccines elicited different cell-mediated immune responses and levels of PCV2d viremia against PCV2d challenge. Although both vaccines used in this study have the same baculovirus system for the expression of the ORF2 subunit of PCV2, each vaccine contained a unique antigen preparation by use of the baculovirus expression system (38). This as well as differences in adjuvant formulation between the vaccines may be as the reasoning behind the differences in protective cell-mediated immunity and induction and reduction of both PCV2 viremia and lymphoid lesions. Further studies are needed to elucidate these differences between the two vaccines.

This comparative study under experimental conditions was the first evaluation between a combination of two monovalent vaccines mixed into a combo prior to vaccination and a ready-to-use bivalent vaccine of PCV2 and *M. hyopneumoniae*. Vaccination remains the most efficient and economical method to-date for the control of PCVAD and enzootic pneumonia. More than 70 percent of Korean pigs receive vaccines to protect against PCV2 and *M. hyopneumoniae* (http://www.kahpha.or.kr) as PCVAD and enzootic pneumonia remain the two most prominent and costly diseases that affect swine. An important advantage in using either a combination of two monovalent vaccines mixed into a combo prior to vaccination or a ready-to-use bivalent vaccine exists as either reduces the number of injections administered to the pig. The results of this study demonstrate a strategic method in controlling two important diseases for swine producers and practitioners.

## Data Availability Statement

All datasets generated for this study are included in the article/supplementary material.

## Ethics Statement

The animal study was reviewed and approved by Seoul national university Instituional Animal Care and Use Committee (SNU-190712-5).

## Author Contributions

SY performance of the experimental trials, data analysis, and writing of the manuscript. TO, KP, and HC preparation of the inoculum and lab analysis. CC development of protocol, design of the study, review of the final manuscript, and approval for publication. All authors read and approved the final manuscript.

## Conflict of Interest

The authors declare that the research was conducted in the absence of any commercial or financial relationships that could be construed as a potential conflict of interest.
